# Frequency, Characteristics, and Correlates of Cognitive Complaints in a Cohort of Individuals with Post-Acute Sequelae of COVID-19

**DOI:** 10.3390/brainsci14010003

**Published:** 2023-12-20

**Authors:** Cayla Muschel, Sean T. Lynch, Rhea Dornbush, Lidia Klepacz, Sivan Shahar, Stephen J. Ferrando

**Affiliations:** 1 Department of Psychiatry and Behavioral Sciences, New York Medical College, Valhalla, NY 10595, USA; cmuschel@mail.yu.edu (C.M.); sean.lynch@mountsinai.org (S.T.L.); lidia.klepacz@wmchealth.org (L.K.); sivan.shahar@mountsinai.org (S.S.); 2 Department of Psychiatry, Westchester Medical Center Health System, Valhalla, NY 10595, USA; rhea_dornbush@nymc.edu; 3 Department of Psychiatry, Icahn School of Medicine at Mount Sinai, Mount Sinai Beth Israel, New York, NY 10003, USA; 4 Department of Psychiatry, Icahn School of Medicine at Mount Sinai, Mount Sinai Hospital, New York, NY 10029, USA

**Keywords:** cognitive complaints, brain fog, COVID-19, depression, neuropsychological performance

## Abstract

Background: Cognitive complaints are among the most frequent symptoms of post-acute sequelae of COVID-19 (PASC). This study aimed to investigate the frequency, characteristics, and clinical correlates of cognitive complaints (CC) in PASC, particularly in relation to objective neuropsychological (NP) performance. Methods: Seventy-four participants underwent psychiatric, medical, and NP testing approximately 7 months after acute COVID-19. The Patient Assessment of Own Functioning Inventory (PAOFI) was used to characterize the frequency and severity of CC in domains of memory, language, and cognitive/executive function. The associations of CC with sociodemographic, medical, psychiatric, and NP variables were assessed utilizing correlational analysis, logistic regression, and pairwise comparisons of those categorized as having CC vs. not having CC. Results: Taken together, approximately one-third of the study participants had clinically significant CC. Memory difficulty was the most frequent CC, although all categories were frequently endorsed. Memory and cognitive/executive complaints correlated with NP tests in these and multiple other NP domains. CC were more likely to be under-reported in those with diminished NP performance than over-reported in those without diminished performance. Acute COVID-19 symptom severity, elevated depressive symptoms, and NP tests of diminished attention and psychomotor processing speed were independent predictors of CC in logistic regression. Conclusions: Cognitive complaints after acute COVID-19 should be taken seriously, as they are likely to reflect diminished NP performance, as well as medical, psychiatric, and functional burdens. However, patients with PASC may not accurately identify or characterize objective cognitive difficulties, so programs offering comprehensive care for patients with PASC should offer formal neuropsychological testing.

## 1. Introduction

Severe acute respiratory syndrome-coronavirus (SARS-CoV-2), the virus that causes COVID-19, has precipitated significant morbidity and mortality in the acute stages. The emerging literature suggest that symptoms outlast acute infection in approximately a third of patients, a condition called post-acute sequelae of COVID-19 (PASC), commonly referred to as “long COVID”. Subjective neuropsychiatric complaints are common in PASC, with fatigue, sleep disturbance, mood changes, and cognitive problems being the most common [1]. The mechanism of brain involvement precipitating neuropsychiatric problems or complaints in PASC patients has not yet been conclusively identified. Persistent systemic and neuro-inflammation triggered by SARS-CoV-2 infection has been highlighted as a likely culprit [2]. Cognitive complaints (CC) in PASC, commonly described by patients as “brain fog”, can include subjective difficulty with memory, focus, mental exhaustion, and executive functioning and frequently co-occur with fatigue, sleep disorders, and psychiatric symptoms. While reported in up to one-third or more of patients with a range of COVID-19 illness severity [3], subjective CC are often not formally characterized in clinical practice. Multidimensional measures such as the Patient’s Assessment of Own Functioning Inventory (PAOFI) [4] may be used to characterize CC. The PAOFI has subscales for memory, language, sensorimotor skills, and higher-level cognition/executive functioning, but it has been applied mostly in research settings.

The relationship between CC and objective neuropsychological (NP) impairment after COVID-19 has not yet been fully elucidated. This is important because clinicians who treat PASC patients need guidance on how to interpret and investigate these complaints. There is substantial literature exploring the relationship of subjective CC to actual NP test performance in other disease entities, including other infectious diseases (i.e., HIV/AIDS and Lyme) [5,6,7], postural orthostatic tachycardia syndrome, patients receiving chemotherapy for breast cancer [8], chronic fatigue syndrome [9], and neurological conditions such as multiple sclerosis [10], among others. Although these syndromes are acquired and managed differently, patients report cognitive dysfunction akin to PASC patients. Thus, research from studies in these disease states may provide insight to guide research into COVID-19-related CC.

Studies on HIV and post-chemotherapy breast cancer patients, including those utilizing the PAOFI, may inform the approach to understanding CC as they relate to NP performance, distress, and disease status after COVID-19. In these disease states, studies on the relationship between CC and objective NP performance yield inconsistent results; complaints can relate to NP performance, measures of distress, or both. Generally speaking, the strongest and most consistent predictors of CC have been depression, anxiety, and other psychiatric conditions, while the relationship to objective NP performance is less consistent [11,12,13,14,15,16].

Preliminary research on CC in COVID-19 patients reflects the mixed results reported in the HIV and post-chemotherapy cancer literature. One study demonstrated that post-COVID patients with CC exhibited deficits in attention, executive function, learning, and long-term memory [17]; others reported that subjective cognitive symptoms were associated with female sex, depression, and PTSD symptoms, but not objective neurocognitive test performance [3]; and yet others found no association between CC and neurocognitive tests or mood [18]. Importantly, studies to date have included heterogeneous, largely clinical populations with differing neurocognitive and psychiatric assessments, and most have not employed standardized measurement of CC, such as the PAOFI. Pihlaja et al. (2023) [3] utilized the A-B Neuropsychological Assessment Schedule (ABNAS) to assess for CC; however, they utilized the Montreal Cognitive Assessment (MoCA) to assess for NP function. This screening test may have limited sensitivity to detect NP impairment in COVID-19 [19].

Our prior work suggested that higher levels of CC on the PAOFI, as well as extremely low NP test performance, were most prominent in patients seeking post-COVID care for “brain fog” compared to those who are not seeking care [20]. Furthermore, we found that extremely low performance was predicted by levels of COVID symptoms (both at the time of acute illness and at the time of assessment months later), levels of depressive symptoms, number of medical comorbidities, and CC as measured by the PAOFI. Based on our preliminary work, we endeavored here to investigate in more depth the relationship between CC, as measured by the PAOFI, domain-specific NP performance, distress, and persistent physical symptoms in a mixed sample of community and clinical PASC patients who experienced mild to moderate COVID-19 illness.

In particular, we aimed to investigate the following:

1. The frequency of cognitive complaints, as measured by the PAOFI, in an ambulatory cohort approximately 7 months after acute COVID-19. Based on prior literature, we hypothesized that clinically significant cognitive complaints, when assessed with a standardized instrument, would be found in approximately one in three study participants.
2. The strength of associations between subjective cognitive complaints on the PAOFI and objective NP performance. In the existing literature, cognitive complaints are associated with depression rather than objective NP performance across multiple clinical populations [11,12,13,14,15,16]. Therefore, we hypothesized that subjective cognitive complaints would be strongly correlated with depression and other measures of distress but weakly or not at all correlated with objective NP test performance.
3. Whether cognitive complaints in specific domains, particularly memory, language and cognitive/executive function, are correlated with performance on NP tests that assess those domains. In studies in which cognitive complaints correlated with reduced NP performance, complaints correlated with impairments both in the corresponding domain and in other domains [15,16]. Therefore, we hypothesized that complaints would not be domain-specific.
4. Correlations and predictors of cognitive complaints after COVID-19 among sociodemographic, medical, psychiatric, and NP variables. We hypothesized that pre-existing psychiatric history, depressive symptoms, and severity of COVID-19 illness would predict cognitive complaints.

## 2. Methods

The data for this study derive from the baseline evaluation of 74 participants enrolled in an ongoing longitudinal investigation of neuropsychological, medical, and psychiatric sequelae of coronavirus infection. Participants were recruited from the Westchester County, New York area via flyers, networking, social media, and referrals from the Westchester Medical Center (WMC) Health System Post-COVID-19 Recovery Program. Prerequisites included aged 20 years or older; (2) documented positive COVID-19 nasopharyngeal or antibody test prior to vaccination; (3) established recovery from acute COVID-19 infection as per Centers for Disease Control and Prevention (CDC) recommendations (10–20 days post symptom onset; 24 h without fever); (4) fluent in English; (5) completed eighth-grade education; and (6) capacity for informed consent. Potential participants underwent telephone screening to determine eligibility. The study excluded individuals with a history of major neurocognitive disorders, intellectual disability, traumatic brain injury with loss of consciousness, or psychiatric instability, in addition to those with uncorrected visual/hearing deficits.

Risks and benefits were discussed with eligible participants, who then signed written informed consent. The study was approved by the New York Medical College Institutional Review Board (Protocol #14400) as well as the WMC Health System Clinical Research Institute on 30 October 2020. Participants first reported their sociodemographic, medical, and psychiatric information via self-report questionnaires. This took approximately 10–15 min. Participants then completed the PAOFI and were interviewed by trained evaluators (S.T.L., S.S.), who subsequently performed and scored a brief testing battery under the supervision of the study principal investigator (S.J.F.) and co-principal investigator (R.D.) (a board-certified neuropsychologist.) Participants received $40 as compensation.

Initial findings suggested that a percentage of individuals who contract COVID-19 demonstrate highly impaired neuropsychological function in multiple cognitive domains and that self-reported cognitive problems were a relatively reliable indicator of cognitive difficulty [20]. As further data were obtained, further analyses were conducted to produce more definitive conclusions.

### 2.1. Study Measurements and Instruments

Sociodemographic measures included age, gender, race, relationship status, years of education, and current employment.

Medical measures included self-reported medical history, including underlying chronic medical comorbidities and a detailed history of COVID-19 symptoms, treatment, and hospitalization, and time since diagnosis. The severity of acute COVID-19 symptoms and severity of PASC symptoms at the time of appointment was measured by a scale adapted from the CDC [21], evaluating the magnitude (absent, mild, moderate, or severe) of 11 COVID-19 symptoms (scale score ranges from 0 to 33). Participants also completed the Lawton–Brody Instrumental Activities of Daily Living Scale (IADL) [22], which analyzes difficulty with practical everyday functioning on a scale of 0–8, and the 11-item Chalder Fatigue Scale [23], which assesses the extent of mental and physical fatigue on a score of 0–33. A score of 21 or greater is considered clinically significant.

Psychiatric variables included the history of substance use or psychiatric illness prior to contracting COVID-19, any psychiatric medications, and self-report questionnaires to gauge current psychiatric status. Self-report questionnaires included the Patient Health Questionnaire-9 (PHQ-9) [24], which evaluates depressive symptoms based on Diagnostic and Statistical Manual for Mental Disorders—5th Edition (DSM-5) major depression criteria; the Posttraumatic Stress Disorder Checklist for DSM-5 (PCL-5) [25]; the Generalized Anxiety Disorder-7 questionnaire (GAD-7) [26]; and the Endicott Quality of Life Enjoyment and Satisfaction Scale (Endicott QLESQ) [27], which assesses overall life satisfaction in 14 areas and has a raw score range of 0–70. Scores on the questionnaires were categorized based on cutoff values in the medical literature. For PHQ-9, a score of ≥10 out of 27 signals clinically significant depressive symptoms; for Generalized Anxiety Disorder-7, a score of ≥ 10 out of 21 signals clinically significant anxiety; and for Posttraumatic Stress Disorder Checklist for DSM-5, a score of ≥33 out of 80 may indicate clinically significant PTSD symptoms.

The neuropsychological battery estimated premorbid function prior to COVID-19 infection (utilizing the Test of Premorbid Function) [28] and assessed specific cognitive domains that have been affected in other clinical populations. These domains included attention; auditory/verbal and visual immediate and delayed memory; visuospatial and constructional abilities; psychomotor speed; language; and executive function. The battery included the Repeatable Battery for the Assessment of Neuropsychological Status (RBANS) [29] Form A (total and 5 subscale scores), the Trail Making Test Parts A and B [30], verbal fluency (letter and category) [31], and the Stroop Color–Word Test [32]. In total, 11 test scores were obtained per participant.

### 2.2. Data Analysis

NP test scores were converted to standardized t-scores and analyzed as continuous measures. In addition, participants were classified into categories of normal, low, and extremely low NP performance. For this, investigators applied accepted clinical practice, in which a score of one or more standard deviations below age-matched population-based normative values on two or more neurocognitive tests qualifies as “Low” performance and for which a score of two or more standard deviations below normative values on two or more tests qualifies as “Extremely Low” performance [33].

Analyses were conducted on the entire sample of 74 participants and on two subgroups—a “cognitive complaint” (CC) group and a “non-complaint” (NC) group. In order to assess for the presence and severity of CC, participants completed the PAOFI, which assesses subjective CC, yielding an average score of 0–5 (0 = almost never; 1 = very infrequently; 2 = once in a while; 3 = fairly often; 4 = very often; and 5 = almost always) for memory, language and communication, handedness, sensory perception, and cognitive/intellectual functioning. For the study, the PAOFI subscales most associated with everyday cognitive functioning, including memory, language, and cognitive/executive functioning, served as measures of subjective CC. For correlational analyses, the PAOFI was used as a continuous measure of severity by calculating the total score on each subscale. To provide an operational definition of clinically significant CC, the study utilized an average score of 3 or above (fairly often, very often, or almost always) on the PAOFI subscales to indicate clinically significant levels of memory, language, and cognitive/executive complaints.

Data were analyzed using SPSS software Version 29 [34]. These included descriptive statistics (frequency, mean, and standard deviation); Chi-square for group comparisons on categorical variables; and independent and one-sample t-tests for group comparisons on continuous variables. Logistic regression was used to identify independent predictors of cognitive complaints on the PAOFI, using as predictors individual NP scores, medical variables, and psychiatric variables that distinguished between complaint and non-complaint groups. Correlative measures were utilized to investigate the potential for domain specificity. Pearson correlations are reported, as well as coefficients of determination, which indicate shared variance between the two variables. Because of the small but well-characterized sample and exploratory nature of the study, a more inclusive approach to minimize type 2 error was utilized, with a significance level of *p* < 0.05 used to determine statistical significance.

## 3. Results

### 3.1. Overall Group Characteristics (Table 1)

The participants had a mean age of 43 years; approximately 70% were female, 65% were White, 65% were in a relationship, and 80% were employed. On average, participants had a college-level education (Table 1).

From a medical standpoint (Table 1), the participants contracted acute COVID-19, on average, 7 months prior to assessment, and 62% of participants were clinical patients seeking post-COVID care. Participants reported, on average, 1.5 comorbid conditions. Reported acute versus current symptoms declined, with general severity in the mild to moderate range; however, 60% (N = 73) of the participants reported continued clinically significant fatigue as measured using the Chalder Fatigue Scale at the time of assessment.

Psychiatrically (Table 1), approximately 42% reported a pre-existing psychiatric history prior to contracting COVID-19. Utilizing cutoff scores, 53% of participants screened positive for clinically significant depression on the PHQ-9, 31% of participants screened positive for clinically significant anxiety on the GAD-7, and 24% of participants screened positive for significant PTSD symptoms on the PCL-5. Twenty-three percent of the overall sample endorsed at least one difficulty with IADL.

Regarding NP results, the Test of Premorbid Function (Table 1) estimated that the sample had a high–normal premorbid intellectual function. However, overall, 59% of the sample had low or extremely low neuropsychological test performance, with 20% being in the extremely low category. Performance on RBANS total, RBANS immediate and delayed memory, RBANS visuospatial/constructional, RBANS Language, Trails A, Trails B, Letter Fluency, and Stroop Color–Word were all statistically significantly below expected population-based norms ([20]), reflecting diminished performance in multiple cognitive domains, including attention, concentration, information processing speed, immediate and remote memory, and executive function.

### 3.2. Overall Frequency and Correlates of Cognitive Complaints (CC)

Based on the study PAOFI cutoff criteria, 23 (31%) participants had significant CC in one or more of the PAOFI subscales (Table 1). The most frequent domain of CC was memory (N = 19, 25%), followed by cognitive/executive function (N = 16, 22%) and language difficulties (N = 8, 11%). Of the 23 with CC, 14 (61%) endorsed problems in more than one area. Table 2 lists the Pearson Correlation Coefficients and coefficients of determination for PAOFI Memory, Language, and Cognitive/Executive scores with scores on continuous measures of distress and NP tests. First, there was a high degree of correlation between the PAOFI subtests (Pearson r = 0.79–0.84, 62–71% shared variance, all *p* < 0.001). There was a moderately high degree of correlation and corresponding coefficients of determination between all PAOFI subscales and all measures of distress (Pearson r = 0.60–0.72, 36–52% shared variance, all *p* < 0.001). There was a low to moderate degree of significant correlation between PAOFI subscales and many, but not all, NP tests, with coefficients of determination for the significant correlations indicating 6–23% of shared variance. Significant correlations frequently crossed domains (i.e., PAOFI Memory complaints correlated with tests of memory, but also multiple other NP test domains). Of note, NP tests of language (RBANS Language, Letter, and Animal Fluency) did not correlate significantly with any PAOFI subscale, including the PAOFI Language subscale.

In terms of alignment between endorsing any clinically significant CC and overall NP performance, participants who had CC had low or extremely low NP performance 78% of the time (Table 1), whereas they had normal NP performance only 22% of the time. Those with low or extremely NP performance were just as likely to have CC (49%) or no CC (51%); however, only 5 of 30 (17%) of participants with normal NP performance endorsed CC, indicating that under-reporting of NP difficulty may be more likely than over-reporting.

### 3.3. Comparison between Those Who Presented with Clinically Significant Cognitive Complaints (CC) to Those with No Cognitive Complaints (NC)

The CC group was significantly older than the NC group but demonstrated no other significant sociodemographic differences (Table 1). The CC group reported significantly more comorbid medical conditions, acute COVID-19 symptoms at the time of infection, current post-COVID symptoms, higher levels of fatigue, and diminished IADLs, and they were more likely to be seeking post-COVID care for PASC.

There were no significant differences between the two groups in previous psychiatric history. However, the CC group presented with significantly higher levels of depressive symptoms on the PHQ-9 and higher levels of anxiety and PTSD symptoms on the GAD-7 and PCL, respectively.

In terms of NP performance, the CC and NC groups were highly similar regarding estimated premorbid intellectual function; however, the CC group performed significantly lower on all parts of the RBANS total score, except for the RBANS Delayed Memory subscale. The CC group also performed significantly lower on the total Stroop Color Word, MoCA, and Trail Making Test Parts A and B.

To identify clinical factors that might predict clinically significant CC, we conducted a backward stepwise logistic regression analysis, including any CC on the PAOFI as the dependent variable (Table 3). The logistic regression model was statistically significant (chi sq = 53.088, df = 5, *p* < 0.001). Peak acute COVID-19 symptom severity, Chalder fatigue score, PHQ-9 score, RBANS attention score, and Trail Making Part A score correctly classified 90.4% of those with CC. All of the variables in the final model, except for the Chalder fatigue score, were independently predictive.

## 4. Discussion

This investigation allowed for a direct comparison of cognitive complaints, as measured using a standardized instrument, the PAOFI, with NP test performance in multiple domains and to investigate medical and psychiatric correlates of CC in the months after recovery from acute COVID-19. The findings appear to have important implications for detecting, evaluating, and treating CC in clinical practice.

In this cohort of individuals seen on average 7 months after acute COVID-19, approximately one-third of participants had clinically significant CC, as defined by operational criteria, indicating difficulty in one or more domains “fairly often”, “very often”, or “all the time”. This is similar to the frequency of CC reported by Pihlaja et al. (2023) [3] in their COVID-19 cohort, utilizing a cutoff > 15 on a different subjective assessment scale, the ABNAS, which measures similar domains to the PAOFI, with the exception of cognitive/executive function. As in prior studies in HIV, breast cancer, and COVID-19 [14,15,16], difficulty with memory was the most frequently endorsed cognitive complaint in this cohort, but memory, cognitive/executive, and language complaints were highly inter-correlated. Memory and cognitive/executive complaints displayed a similar pattern of correlation with NP tests measuring those domains, but also other domains as well. For instance, memory complaints were correlated with immediate and delayed memory, but also tests of attention/concentration, processing speed, and executive function. The high degree of inter-correlation reflects the general pattern of diminished NP performance across NP domains in this cohort and suggests that individuals who are endorsing problems in memory or executive function are likely to have diminished performance in multiple other domains. In addition, these cognitive domains are not discrete, such that memory tasks may require elements of executive function and vice versa.

In contrast to memory and cognitive/executive complaints, language complaints on the PAOFI did not correlate significantly with any NP test of language employed in this study. Language complaints did correlate to a mild to moderate degree with tests of attention, memory, processing speed, and executive function. This may indicate that individuals who are perceiving language difficulty after COVID-19 may be misattributing problems they are experiencing in other cognitive domains. It is also possible that the NP tests of language employed in this study were not sensitive to the language problems experienced by these individuals.

Another interesting finding from this study is that individuals who endorse clinically significant CC, as operationalized in this cohort, most often (78% of the time) *do* have some degree of diminished NP performance. However, approximately one out of five individuals who endorsed CC did not have diminished NP performance, which may indicate other factors such as distress are leading to magnification of cognitive difficulty for some individuals. Perhaps more noteworthy, over half of the individuals who had low or extremely low NP performance did not have clinically significant CC, as operationalized in this study. This suggests that there may be a significant proportion of the post-COVID population that may underestimate or not recognize diminished NP performance. This phenomenon of cognitive anosognosia is common among people with cognitive impairment and appears to increase with the severity of impairment [35,36]. Taken together, these findings indicate that the clinical use of the PAOFI as a screening tool and/or a measure of severity, may have limitations. In particular, the cutoffs used in this study may yield false negatives (i.e., individuals with diminished NP performance and little or no CC) and a smaller number of false positives (i.e., individuals with CC without diminished performance). A less stringent cutoff of 2 “occasional” was considered; however, this cutoff was not regarded as face-valid for clinical use and, in fact, significantly elevated the false positive rate without substantially increasing the detection of true positives. In either case, the findings indicate that clinical use of a standardized measure of CC will complement, but cannot substitute for, clinical observation, collateral information-gathering from supportive others, and formal testing of patients where there is clinical suspicion, particularly to detect those individuals who are under-reporting CC.

In this cohort, participants with CC had multiple differences across medical, psychological, and NP variables compared to those with NC, indicating a greater degree of medical, psychological, and NP difficulty among those with CC. The only sociodemographic variable that differed between those with CC and NC was age, with those having CC being older. Furthermore, correlations between individual CC and individual variables in medical, psychiatric, and NP domains identified a clear hierarchical relationship. In general, the strongest, most significant correlations were between CC and subjective measures of psychiatric distress, followed by medical symptoms (acute and chronic post-COVID symptoms, fatigue, IADL difficulty) and then, age and NP tests, which bore the weakest significant correlations. These findings are not surprising because associations of CC across medical illnesses, including HIV, post-chemotherapy breast cancer, and previous studies of COVID-19 indicate the most consistent associations are between CC and measures of distress, particularly depression, while significant associations are less consistently found between CC and measures of medical severity and objective NP test performance [3,11,12,13].

In an effort to develop a predictive model of CC in the setting of multiple significant cross-sectional associations, logistic regression was conducted, which correctly categorized the participants >90% of the time. Measures from each measurement domain, with the exception of sociodemographic, were included in the final model, and may help to establish an “at risk” profile for CC after COVID-19. The severity of acute COVID-19 illness was the most significant independent predictor of CC in the cohort, indicating that the severity of acute illness is important to perceived complaints even months after the illness. We have previously identified acute COVID-19 severity to be a predictor of NP test performance months after illness [20], which now extends to CC. The severity of current depressive symptoms is also a significant predictor of CC, which is consistent with prior research on COVID-19, as well as HIV, post-chemotherapy breast cancer, and other illnesses [3,11,12,13,14,15]. The relationship of depression to CC and NP performance is known to be complex [37]. Difficulty with attention and concentration are symptoms of depression, and depression is associated with NP deficits in multiple domains, including attention, concentration, working memory, processing speed, and executive function [38,39]. The CC, as well as the NP difficulties of participants in this study are highly consistent with this profile. Importantly, diminished performance on objective tests of attention and speed of processing, RBANS Attention, and Trail Making Test Part A were also independent predictors of CC in this cohort, which has been previously reported in HIV infection [14,15].

There are a number of significant limitations to this study. The cohort was relatively small, and assessments were cross-sectional. Multiple comparisons were conducted, and the investigators chose not to correct for multiple comparisons so as to minimize Type 2 error. It is noteworthy, however, that the cohort was very carefully characterized, including in-person assessment with a standardized assessment of CC, the PAOFI. In addition, the majority of significant findings were at the level of *p* < 0.01 or *p* < 0.001. Further, findings were consistent with clinical experience in treating the post-COVID population [40]. Formal clinical psychiatric interviews were not conducted, so psychiatric diagnosis could not be determined. There is also potential for response bias, namely that participants might over-report or under-report the magnitude to which they experienced fatigue, psychiatric symptoms, or subjective cognitive impairment. This could be motivated by social desirability or acquiescence bias [41]. Finally, while regression analysis provided for a predictive model of CC in the months after COVID-19, causal associations cannot be determined, as participant ratings of CC, distress, and medical burden were carried out concurrently. A possible exception to this is COVID-19 symptom severity at the time of acute illness; however, this rating was carried out retrospectively and may have been subject to recall bias. Only longitudinal analysis can begin to address causal associations between CC after COVID-19 and medical, psychiatric, and NP variables. Because of this limitation, the mechanism by which PASC may contribute to neuropsychological deficits and/or cognitive complaints remains unclear.

## 5. Conclusions

Despite the limitations cited above, the results of this study may have significant clinical relevance for practitioners treating patients with cognitive complaints in the months after COVID-19 illness. First, cognitive complaints should be taken seriously, as they may reflect actual difficulty with neuropsychological status as well as depression, ongoing medical symptom burden, and functional status. Second, even when a standardized instrument is utilized to characterize cognitive complaints, such as the PAOFI, patients may not be accurate in either detecting the presence or absence of NP difficulty or in the nature of this difficulty if endorsed. In fact, in this cohort, there was a significant risk that participants under-reported, rather than over-reported, cognitive difficulty that was detected on NP testing. While this would suggest that cognitive screening is necessary for patients with PASC, the issue is complicated by the fact that typical neurocognitive screening tests, such as the MoCA, are unreliable in detecting mild to moderate NP difficulty in COVID-19 [19]. In this study, the MoCA score was not a significant predictor of cognitive complaints in the regression model. Thus, in clinical populations of patients with PASC, clinicians should maintain a high index of suspicion, and NP assessment should be offered, even in the absence of cognitive complaints. The NP assessment battery utilized in this study may be useful for clinical application, as it provides an estimate of pre-morbid function, assesses key NP domains, and takes approximately 45–60 min to administer. Finally, one can only speculate on the complex causal relationships between cognitive complaints, objective NP performance, distress, and ongoing medical symptom burden after COVID-19. Longitudinal studies are sorely needed to characterize the durability of cognitive complaints and to clarify causal associations so as to guide treatment intervention research.

## Figures and Tables

**Table 1:** Description of the entire cohort and comparisons of participants with no cognitive complaints to those with cognitive complaints across sociodemographic, medical, psychiatric, and neuropsychological domains.

Measure	Total Sample (*n* = 74)	No Cognitive Complaints (*n* = 51)	Cognitive Complaints (*n* = 23)	Statistic, df, *p* Value
Sociodemographic Characteristics				
Age (m, SD)	43.49 (15.06)	40.39 (14.77)	50.35 (13.61)	t = −2.748; df = 72; *p* = 0.008
Female (N, (%))	52 (70%)	35 (69%)	17 (74%)	Chi sq = 0.212, df = 1, *p* = 0.65
Ethnic Minority (N (%))	26 (35%)	17 (33%)	9 (39%)	Chi sq =0.234, df 1, *p* = 0.63
Years of Education (m, SD)	16.05 (2.20)	16.31 (2.13)	15.48 (2.27)	t = 1.529, df 72, *p* = 0.13
In a Relationship	48 (65%)	34 (67%)	14 (61%)	Chi sq = 0.23, df 1, *p* = 0.6229
Employed or Student	59 (80%)	42 (82%)	17 (74%)	Chi sq = 0.70, df = 1, *p* = 0.40
Medical Characteristics				
Days between acute illness and assessment (m, SD)	222.4 (134.3)	208.90 (136.3)	251.04 (127.9)	t = −1.25; df = 72; *p* = 0.21
# of medical comorbidities (m, SD)	1.53 (1.50)	1.25 (1.34)	2.13 (1.69)	t = −2.40; df = 72; *p* = 0.019
Seeking medical care for PASC (N, %)	46 (62%)	26 (51%)	20 (87%)	Chi sq = 8.72, df = 1, *p* = 0.003
Peak COVID symptoms (m, SD)	16.64 (6.24)	14.45 (5.48)	21.48 (5.03)	t = −5.235; df = 72; *p* < 0.001
Appt 1 COVID symptoms (m, SD)	6.45 (4.78)	4.98 (4.15)	9.7 (4.55)	t = −4.391; df 72; *p* < 0.001
Chalder Fatigue Scale, (N = 73) (m, SD)	21.73 (7.66)	N = 50, 19.22 (7.48)	N = 23, 27.17 (4.69)	t = −5.521, df 64.211, *p* < 0.001
Chalder criteria for clinically significant fatigue (N = 73) (N, %)	44 (60.3%)	22 (43%)	22 (95.7%)	Chi sq = 18.141, df 2, *p* < 0.001
Psychiatric Characteristics				
Prior psychiatric history (N, %)	31 (42%)	18 (35%)	13 (57%)	Chi sq = 2.934, df 1, *p* = 0.087
PHQ−9 Score (m, SD)	10.2 (6.19)	7.82 (5.30)	15.48 (4.58)	t = −5.985; df 72; *p* < 0.001
Met PHQ−9 criteria for clinically significant depression (N, %)	39 (53%)	18 (35%)	21 (91%))	Chi sq = 19.949, df 1, Fisher’s exact *p* < 0.001
GAD, (m, SD)	7.35 (5.51)	5.20 (4.10)	12.13 (5.29)	t = −6.138, df 72; *p* < 0.001
Met GAD−7 criteria for clinically significant anxiety (N, %)	23 (31%)	8 (16%)	15 (65%)	Chi sq = 18.155, df 1, Fisher’s exact *p* <0.001
PCL−5 score (m, SD)	M = 21.81 SD = 15.202	M = 16.12 SD = 11.554	M = 34.43 SD = 14.890	t = −5.757; df 72; *p* < 0.001
Met PCL- 5 criteria for clinically significant PTSD symptoms (m, SD)	18 (24%)	4 (8%)	14 (61%)	Chi sq = 24.213 Df = 1, Fisher’s exact *p* <0.001
IADL (m, SD)	N = 73 7.48 (1.14)	N = 50 7.76 (0.77)	N = 23 6.87 (1.55)	t = 2.616, df = 27.158, *p* = 0.014
Neuropsychological Characteristics				
TOPF, (m, SD)	109.51 (12.06)	109.59 (13.14)	109.35 (9.48)	t = 0.079; df 72; *p* = 0.937
Normal NP Performance (N, %)	30 (41%)	25 (49%)	5 (22%)	
Low or Extremely Low NP performance (N, %)	44 (59%)	26 (51%)	18 (78%)	Chi sq = 4.894 df = 1, *p* = 0.027
Extremely Low NP Performance only (N, %)	15 (20%)	8 (16%)	7 (30%)	Chi sq = 2.133 df = 1 Fisher’s exact *p* = 0.211
RBANS Total, (m, SD)	93.65 (4.21)	97.88 (11.97)	84.26 (14.52)	t = 4.235, df = 72, *p* < 0.001
RBANS Immediate Memory, (m, SD)	88.57 (16.54)	92.73 (14.33)	79.35 (17.67)	t = 3.453, df 72, *p* < 0.001
RBANS Visuospatial, (m, SD)	104.95 (16.38)	108.67 (12.60)	96.7 (20.65)	t = 2.573 df = 29.634 *p* = 0.015
RBANS Language, (m, SD)	M = 94.20 SD = 14.387	M = 96.92 SD = 15.324	M = 88.17 SD = 9.898	t = 2.507, df 72, *p* = 0.014
RBANS SMF				t = 3.950, df = 72, *p* < 0.001
RBANS Attention, (m, SD)	97.39 (15.34)	101.08 (14.70)	89.22 (13.74)	t = 3.277, df 72, *p* = 0.002
RBANS Delayed Memory, (m, SD)	92.16 (15.71)	94.29 (14.41)	87.43 (13.74)	t = 1.763, df 72, *p* = 0.082
Trails A, (m, SD)	46.30 (11.16)	48.2 (11.16)	42.09 (10.17)	t = 2.238, df 72, *p* = 0.028
Trails B, (m, SD)	44.51 (11.39)	M = 46.86 SD = 10.214	M = 39.30 SD = 12.327	t = 2.760, df 72, *p* = 0.007
Letter Fluency, (m, SD)	N = 73 47.30 (10.58)	N = 50 47.74 (10.63)	46.35 (9.213)	t = 0.541, df = 71, *p* = 0.590
Category Fluency, (m, SD)	48.64 (10.58)	49.31 (10.97)	47.13 (9.71)	t = 0.820, df = 72, *p* = 0.415
Stroop CW, (m, SD)	47.169 (11.61)	49.29 (11.77)	42.46 (9.92)	t = 2.420, df= 72, *p* = 0.018
Total MOCA, (m, SD)	25.74 (2.65)	26.39 (2.65)	24.30 (2.06)	t = 3.351, df = 72, *p* = 0.001

**Table 2:** Pearson correlation coefficients for the associations of PAOFI Memory, Language, and Cognitive/Executive scores with scores on continuous measures of distress and neuropsychological tests.

	PAOFI Domain	Memory	Language	Cognitive/Executive
Measurement Domain				
Cognitive Complaints				
PAOFI Memory		-	0.79 (r^2^ = 0.62) ^1^	0.84 (r^2^ = 0.71) ^1^
PAOFI Language		-	-	0.79 (r^2^ = 0.62) ^1^
PAOFI Cognitive/Executive		-	-	-
Sociodemographic				
Age		0.28 (r^2^ = 0.08) ^3^	0.13 (r^2^ = 0.02) ^4^	0.31 (r^2^ = 0.10) ^2^
Psychiatric				
PHQ-9		0.68 (r^2^ = 0.46) ^1^	0.63 (r^2^ = 0.40) ^1^	0.72 (r^2^ = 0.52) ^1^
GAD-7		0.63 (r^2^ = 0.40) ^1^	0.62 (r^2^ = 0.38) ^1^	0.60 (r^2^ = 0.36) ^1^
PCL-5		0.65 (r^2^ = 0.42) ^1^	0.60 (r^2^ = 0.36) ^1^	0.61 (r^2^ = 0.37) ^1^
Medical				
Acute COVID symptoms		0.48 (r^2^ = 0.23) ^1^	0.45 (r^2^ = 0.20) ^1^	0.45 (r^2^ = 0.20) ^1^
Appt. 1 COVID symptoms		0.57 (r^2^ = 0.32) ^1^	0.58 (r^2^ = 0.34) ^1^	0.62 (r^2^ = 0.38) ^1^
Chalder Fatigue Scale		0.64 (r^2^ = 0.41) ^1^	0.66 (r^2^ = 0.44) ^1^	0.66 (r^2^ = 0.44) ^1^
IADL		−0.41 (r^2^ = 0.17) ^1^	−0.28 (r^2^ = 0.08) ^3^	−0.53 (r^2^ = 0.28) ^1^
Neuropsychological				
TOPF		−0.04 (r^2^ = 0.002) ^4^	−0.06 (r^2^ = 0.004) ^4^	−0.07 (r^2^ = 0.005) ^4^
RBANS Total Scale		−0.45 (r^2^ = 0.20) ^1^	−0.36 (r^2^ = 0.13) ^2^	−0.39 (r^2^ = 0.15) ^1^
RBANS Immediate Memory		−0.34 (r^2^ = 0.12) ^2^	−0.32 (r^2^ = 0.10) ^2^	−0.36 (r^2^ = 0.13) ^1^
RBANS Visuospatial/Constructional		−0.38 (r^2^ = 0.14) ^1^	−0.29 (r^2^ = 0.08) ^3^	−0.32 (r^2^ = 0.10) ^2^
BBANS Delayed Memory		−0.29 (r^2^ = 0.08) ^3^	−0.21 (r^2^ = 0.04) ^4^	−0.26 (r^2^ = 0.07) ^3^
RBANS Language		−0.19 (r^2^ = 0.04) ^4^	−0.18 (r^2^ = 0.03) ^4^	−0.13 (r^2^ = 0.02) ^1^
RBANS Attention		−0.39 (r^2^ = 0.15) ^1^	−0.28 (r^2^ = 0.08) ^3^	−0.30 (r^2^ = 0.09) ^2^
Trails A		−0.27 (r^2^ = 0.07) ^3^	−0.18 (r^2^ = 0.03) ^4^	−0.25 (r^2^ = 0.06) ^3^
Trails B		−0.37 (r^2^ = 0.14) ^1^	−0.32 (r^2^ = 0.10) ^2^	−0.32 (r^2^ = 0.10) ^2^
Letter Fluency		−0.11 (r^2^ = 0.01) ^4^	−0.05 (r^2^ = 0.003) ^4^	−0.07 (r^2^ = 0.005) ^4^
Animal Fluency		−005 (r^2^ = 0.003) ^4^	−0.06 (r^2^ = 0.004) ^4^	−0.11 (r^2^ = 0.01) ^4^
Stroop Color/Word		−0.44 (r^2^ = 0.19) ^1^	−0.30 (r^2^ = 0.09) ^2^	−0.39 (r^2^ = 0.15) ^1^
MOCA		−0.50 (r^2^ = 0.25) ^1^	−0.32 (r^2^ = 0.10) ^2^	−0.48 (r^2^ = 0.23) ^1^

^1^ *p* < 0.001; ^2^ *p* < 0.01; ^3^ *p* < 0.05; ^4^ *p* = Not statistically significant.

**Table 3:** Multivariate logistic regression with backward elimination predicting cognitive complaints vs. no cognitive complaints ^1^.

Variable	Odds Ratio	Wald	B	95% Confidence Interval (Lower Bound)	95% Confidence Interval (Upper Bound)	*p*-Value
Peak COVID symptom score	1.321	6.622	0.279	1.069	1.634	0.010
PHQ-9 score	1.363	5.604	0.309	1.055	1.760	0.018
RBANS Attention	0.912	4.679	−0.092	0.840	0.991	0.031
Trails A	0.890	3.967	−0.116	0.794	0.998	0.046
Age	Removed by backward stepwise (conditional) elimination					
Number of Medical Comorbidities						
Appt. 1 COVID symptom score						
Chalder score						
PCL-5 Score						
GAD-7						
RBANS immediate memory score						
RBANS Visuospatial						
RBANS Language						
Trails B						
Stroop CW						
MoCA						

^1^ In order to determine predictors of cognitive complaints, a backward conditional variable selection logistic regression model was developed utilizing all variables with significant differences between the CC group and the NC group. The procedure excluded 12 of the variables and included Chalder in the final model, although it was not statistically significant. The model, including the remaining four predictors (severity of acute COVID-19 illness, PHQ-9 score, RBANS Attention, and Trails A), was significant (−2log likelihood = 37.884, Chi-Square = 53.088, df = 5, *p* < 0.001).

## Data Availability

The data presented in this study are available on request from the corresponding author. The data are not publicly available due to the fact that this is a longitudinal study and data collection is ongoing.
